# Developing and analysing a curriculum map in Occupational- and Environmental Medicine

**DOI:** 10.1186/1472-6920-10-60

**Published:** 2010-09-14

**Authors:** Inga Hege, Dennis Nowak, Stefanie Kolb, Martin R Fischer, Katja Radon

**Affiliations:** 1 Medical Education Unit, University Hospital Munich, Medizinische Klinik - Innenstadt, Ziemssenstr. 1, D-80336 Munich, Germany; 2 Institute and Outpatient Clinic for Occupational, Social and Environmental Medicine, Ludwig-Maximilians-University, Ziemssenstr. 1, D-80336 Munich, Germany; 3 Institute for Learning and Educational Research in the Health Sciences, Private University Witten/Herdecke gGmbH, Germany

## Abstract

**Background:**

During the last 5 years a fundamental curriculum reform was realized at the medical school of the Ludwig-Maximilians-University. In contrast to those efforts, the learning objectives were not defined consistently for the curriculum and important questions concerning the curriculum could not be answered. This also applied to Occupational and Environmental Medicine where teachers of both courses were faced with additional problems such as the low number of students attending the lectures.

The aims of the study were to develop and analyse a curriculum map for Occupational and Environmental Medicine based on learning objectives using a web-based database.

Furthermore we aimed to evaluate student perception about the curricular structure.

**Methods:**

Using a web-based learning objectives database, a curriculum map for Occupational and Environmental Medicine was developed and analysed. Additionally online evaluations of students for each course were conducted.

**Results:**

The results show a discrepancy between the taught and the assessed curriculum. For both curricula, we identified that several learning objectives were not covered in the curriculum. There were overlaps with other content domains and redundancies within both curricula. 53% of the students in Occupational Medicine and 43% in Environmental Medicine stated that there is a lack of information regarding the learning objectives of the curriculum.

**Conclusions:**

The results of the curriculum mapping and the poor evaluation results for the courses suggest a need for re-structuring both curricula.

## Background

The concept of curriculum mapping was developed in the 1980s by English, who defined curriculum mapping as a reality-based record of the content actually taught, how long it was taught, and the match between what was taught and what was assessed [1]. This approach was expanded in the 1990s by Jacobs, who included a timeline, scheduling the taught content within the curriculum, a review of the data, content of exams and an electronic collection of the curriculum data, creating the curriculum map [2].

Harden describes a curriculum map as a map which displays what is taught, how, and when and with which kind of measurements success can be assessed. The logical arrangements of all parts of the curriculum can be made visible. To create a curriculum map is a time consuming task but it offers many opportunities for all stakeholders in a curriculum. For example, it can help teachers to match their course to the overall curriculum, create valid examinations, and help students identify the learning objectives they have to achieve [3].

Over the past 5 years, a fundamental curriculum reform has been realized at the medical school of the Ludwig-Maximilians-University (LMU) as in many other medical schools worldwide [4].

In addition to reorganization of course content, the integration of "new" teaching methods, such as problem-oriented learning (PBL) and E-learning, have been increased [5]. However, learning objectives have so far not been defined consistently for the content domains of the medical curriculum (MeCuM) and a curriculum map had not been created.

Although studies show variant results concerning the effects of learning objectives in increasing learning success and extended memory, they are widely accepted as a necessary component of curriculum planning and in the instructional design process [6,7]. Therefore, many departments like the Institute of Occupational (OM), Social and Environmental Medicine (EM) have made a catalogue of learning objectives for their courses, available to their students (e.g. as a pdf file). But these separate and multiform catalogues are often too extensive and do not allow those in charge of the curriculum to reach conclusions about the overall consistency of the curriculum. This lack of consistency makes it difficult to answer the following questions:

• What are the priorities of the curriculum?

• Are all of the relevant learning objectives covered? If not, which ones are not covered?

• How are learning objectives incorporated into the curriculum?

• Do redundancies within and between content domains exist. If so, are they intended or just planning errors?

• When and how are the assessed learning objectives taught?

The aims of the study were to develop and analyse the curriculum map in occupational (OM) and environmental (EM) medicine based on learning objectives using a web-based application [8,9]. At the same time, an evaluation was implemented to identify views on the curriculum from a student's perspective.

## Methods

A curriculum map for OM and EM based on learning objectives was developed. The reasons for choosing these two content domains as first step of the curriculum mapping were the manageable number of lecturers, the interdisciplinarity of the content and the high interest and enthusiasm of the teachers.

In order to develop the map the following process was undertaken:

1. Survey of the current curriculum structure

2. Attend lectures and tutorials and work through the virtual patient cases during this term to define the learning objectives and course prerequisites in close cooperation with the lecturers.

3. Implement an online evaluation (see appendix 1) of the two curricula by the students at the end of the term

4. Determine the examined learning objectives of the final multiple choice exam

Analyse data

### 1 and 2

The course structure and the learning objectives in OM and EM were surveyed by one person attending the lectures and tutorials during one term. Each of the lectures and tutorials was attended, the learning objectives written down and sent to the lecturer for approval. The online virtual patient cases, a mandatory part of the curriculum, were worked through and tagged with learning objectives. In addition to the learning objectives, data collection included the date and duration of the lecture or tutorial, name of the teacher and number of participants.

### 3: Online evaluation by the students

The evaluation was done by implementing a 36 item Online questionnaire (Additional file 1) that included general questions (age, gender), whether the student attended lecture and why (or why not), information deficits (if any) concerning the curriculum and learning objectives, the students' evaluation of the course concerning overall rating, structure, content, interactivity, tutors and final exam. In addition to these questions on a 5-item Likert scale (1 = totally disagree, 5 = totally agree), a comment field was provided.

The survey items were developed on the basis of the questionnaire designed for the LMU medical curriculum (MeCuM) course evaluations. Students where invited by email after the final exams and reminded twice to complete the anonymous survey.

The results of the questionnaire were analysed in SPSS (SPSS.inc; Version 2.0; IBM) and are presented as mean values with standard deviation. Differences between groups were evaluated using a t-test.

### 4: Learning objectives database

The collected learning objectives were entered into a web-based database, a tool to develop a curriculum map based on specific learning objectives and standard catalogues of learning objectives [8-10]. The objectives were based on the definitions of learning objectives given by Mager *"An objective is a description of a performance you want learners to be able to exhibit before you consider them competent. An objective describes an intended result of instruction, rather than the process of instruction itself." *[11] along with the key concepts found in the Mager model [12]. This model recommends that learning objectives shall fulfil the following three requirements:

• Learning objectives should have a measurable verb.

• Learning objectives should give a specification about what the learners are taught.

• Learning objectives should provide criteria for success and competence shall be defined.

**Figure 1:**
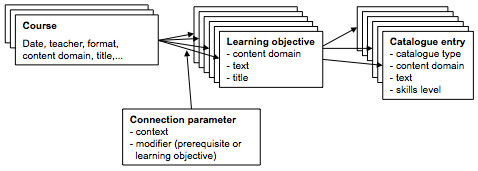
**Model of the learning objectives mapping**.

The web-based tool provides the following functionalities:

Teachers can enter their course and exam data including metadata, i.e. title and duration of lecture, specific learning objectives and prerequisites. The learning objectives and requirements have to be connected to one or more standard catalogue entries and assigned to courses using connection parameters such as context, time and quantification. A model of the mapping mechanism is shown in figure 1. It illustrates how learning objectives and prerequisites, which are connected to catalogue entries, are attached to courses.

The planners of the curriculum can analyze the mapped curriculum and deduce inconsistencies and improvements.

Currently the inconsistencies that can be deduced include:

• Learning objectives that were not covered.

• Overlap with other content domains can be detected.

• Learning objectives covered multiple times can be determined.

• Timing inconsistency (e.g. a prerequisite has not been taught before the course where it is required) can be found.

• Inconsistencies between the learning objectives and the exam content can be identified.

Learners will be given access to the database after the mapping is completed.

The underlying catalogue is an integrated version of the "Hamburger Lernzielkatalog" (HLZK) [13], which is based on the Swiss Catalogue of Learning Objectives [14]. The HLZK consists of 130 general and 3773 particular entries, which define the core curriculum without elective courses. 27 entries are defined in OM, 18 in EM.

In addition to the collection and analysis of the learning objectives, notes have been taken about the usability and useful improvements of the tool.

Because the adequacy of the underlying catalogues was not verified at the beginning of this study, this was also an aspect for investigation.

## Results

### Structure of the OM and EM curricula

211 students (3rd year) participated in OM and 223 students (4^th ^year) in EM. The main aspects of the two curricula including course settings, covered topics and attendance are shown in Additional file 2.

### Results of the students' evaluation

The response was reasonable with 71 responses (33.6%) in OM and 64 (28.7%) in EM. When students were asked for the reasons why they did not attend lectures, 40% in OM while 39% in EM stated that the time of the lecture was inconvenient; 43.5% in EM vs. 16% in OM stated that lack of time was their reason for not attending the lectures.

The students also stated that the subject was valued as "not relevant/boring" (31% OM, 9% EM) while 53% in OM and 43% in EM answered that there is a lack of information regarding the learning objectives.

The overall rating of the different courses indicated a slightly better score for the online cases than for lectures or the tutorial (Additional file 2).

**Figure 2:**
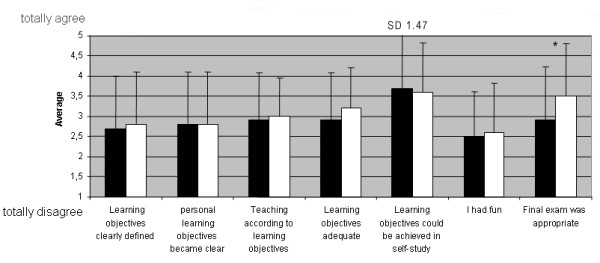
**Results of the online questionnaire**. OM(black): n = 71, EM(white): n = 64, presented are mean values with standard deviation (SD). *p < 0.05.

The results of the questions about the learning objectives of the courses are shown in figure 2.

The results for OM and EM did not differ significantly except for the rating of the final exam. The final exam in EM was rated statistically significantly more appropriate than in OM (p = 0.048).

### Results of the curriculum map analysis

Comparing the learning objectives covered by the curriculum to the underlying catalogue (HLZK), it was shown that in OM, 8 catalogue entries were neither covered in a lecture, nor in a tutorial or case. For EM 8 catalogue entries were not covered.

In OM, the tool identified overlaps with catalogue entries in internal medicine (11), prevention (10) and hygiene (3). In EM overlaps with hygiene (6), clinical chemistry (5) and occupational medicine (4) were identified.

Analysing the redundancies within each curriculum, 31 learning objectives were covered multiple times in OM and 6 in EM. Timing inconsistency could not be discovered within each curriculum. The analysis of the objectives that were examined in comparison with the taught learning objectives, identified some discrepancies. Five learning objectives in OM and two in EM have been part of the MC exam but were not covered in the taught curriculum. Table 1 shows a summary of the collected data.

**Table 1:** Summary of the curriculum analysis

	OM*	EM*
Catalogue entries (HLZK)	27	18
Catalogue entries not covered	8	8
Overlap with other content domains	Internal medicine (11)	Hygiene (6)
	Prevention (10)	Clinical chemistry (5)
	Hygiene (3)	OM (4)
Redundancies within content domain	31 (2-7) times	6 (2-7 times)
Timing inconsistencies	None	None
Tested but not taught learning objectives	5	2

*OM = Occupational Medicine, EM = Environmental Medicine

## Discussion

The results of the study showed that it was feasible to develop and analyse a curriculum map of learning objectives with the tool we created. The analysis of the data identified overlaps, missing learning objectives and discrepancies between the taught and the tested curriculum.

To avoid assessing a "fictional curriculum" [15], which covers what is assumed the students are learning, the survey of the learning objectives was done by one independent person (neither a participating student, nor the lecturer himself), who validated the data with the lecturer. Thereby we believe we have mapped the taught curriculum as well as the tested curriculum, by surveying the exams. To validate this approach it will be necessary to implement future studies with students and lecturers assessing the learning objectives themselves and compare these outcomes with the learning objectives assessed independently. However, as Harden [3] recommended, it will be important to familiarize teachers and students with the use of the map and involve them in the update process.

The analysis of the mapped curricula in combination with the evaluation results and the low attendance rate at the lectures suggest a need for modification. These modifications such as reducing redundancies and adding the not-covered learning objectives to lectures cannot yet be done automatically by the tool, but have to be discussed and agreed upon by the teachers in OM and EM. The aims of such modifications include enhancing the number of participants in the lectures, enhancing motivation of students and sharpening the focus of the teaching.

The response rate of the online survey was reasonable (34% in OM, 29% in EM), but only 16% of the students in OM and 7% in EM actually attended and evaluated the lectures. Therefore the rating of the lectures might change significantly if the attendance rates were higher.

In OM, the tutorials have been rated unsatisfactory and the structure will be reorganised and improved. Due to the low overlapping of content between the tutorials, taught by different teachers, the taught content is not consistent enough to be part of the final exam. Either the content should be changed, which might be difficult due to the different backgrounds of the tutors or the focus of the course could be sharpened. One possibility might be to accept that the tutorials are not exam relevant and deal with special topics in each tutorial, allowing students to elect which tutorials they would like to attend. If the focus of the curriculum includes practical topics such as history taking, physical exam, workplace investigations, it might be useful to include these in tutorials.

On the other hand, the online cases have been well received by the learners, which is confirmed by earlier studies [16]. To increase priority and enhance motivation of students, a strategy could be adapted to increase case-use by integrating exam-relevant cases [17]. This enables all learning objectives covered in the cases to be also exam topics.

The analysis of the not covered learning objectives strongly depends on the defined catalogue entries. Therefore, the completeness and adequacy of the underlying catalogues is essential for the significance of the analysis results. With the low number of defined learning objectives in OM and EM it is evident that the catalogue needs to be extended and modified. For example, work-related accidents are not mentioned in the curriculum or in the catalogue. In both, the practical aspects of OM and EM, such as taking an occupational/environmental history are absent. On the other hand, experts might consider deleting some other non-relevant catalogue entries, e.g. metal fume fever.

The analysis of the curriculum showed redundancies in OM and EM of 5%, though the expected percentage was higher due to the fact that the topics covered are often similar. An explanation for this could be that the lecturers dealt with the same topic but focused on different aspects. Nevertheless it is reasonable to discuss combining OM and EM and teaching both in 4^th ^year. This might also allow tutors to increase the knowledge they require students to know from other content domains like internal medicine (which is currently taught in the same year as OM). Furthermore this might reduce redundancies between OM/EM and other content domains, although both fields have interdisciplinary aspects. Before assessing timing inconsistencies relating to the overall curriculum, the mapping of the relevant content domains like internal medicine has to be completed.

Although the adequacy of the final exam in OM was rated 2.9, the learning objectives for 5 out of 30 questions had not been taught. Having a mapped curriculum provides a review about the learning objectives of the curriculum and thus allows exam preparation to be in alignment with the covered learning objectives. The questions do not necessarily have to be created by the lecturers themselves. In the future one person can design the exam based on the curriculum database.

## Conclusions

The implemented curriculum map in OM and EM was the starting point for the complete mapping of the LMU medical curriculum (MeCuM). Lessons learned from this first step will be considered when extending the mapping and analysis to all MeCuM courses and learning resources.

For example we discovered a need to extend the software. Instead of keeping the knowledge levels attached to the catalogue entries, another connection level will be included to allow teachers to alter the skills level of the catalogue entry. Also it has been detected that the underlying catalogues are not necessarily adequate for all content domains and have to be modified by experts. The next step will be to discuss the necessary adaptations of the HLZK in coordination with specific OM and EM catalogues. It will be important to find and implement solutions for adding catalogue entries while doing editorial checking. Based on these changes in catalogues, the analysis will be repeated.

Currently the re-structuring in OM and EM and necessary changes are discussed and considered in a group of lecturers and students. These changes will be implemented, evaluated and students given access to the database. Acceptance and influences on students learning will be evaluated.

After having implemented modifications, the mapping, evaluating and analysing of both curricula will be repeated and results compared with the data gained so far.

The results from this analysis will be considered when restructuring both curricula. As a second step experiences from this approach will be used to map the complete medical curriculum at the LMU Munich (MeCuM).

## Competing interests

The authors declare that they have no competing interests.

## Authors' contributions

IH drafted the manuscript, developed the underlying software and contributed significantly to the conception, design and implementation of the study.

DN contributed substantially to the conception, design and implementation of the study.

KR and SK contributed significantly to the conception, design and implementation of the study, as well as to the interpretation of data.

MF contributed substantially to the conception and design of the study and gave major didactical inputs.

All authors have been involved revising the manuscript critically and given final approval.

## Pre-publication history

The pre-publication history for this paper can be accessed here:

http://www.biomedcentral.com/1472-6920/10/60/prepub

## Supplementary Material

Additional file 1 **Translated questionnaire developed for this study**. Click here for file

Additional file 2 **Table S1**. Click here for file
