# Supplementary material for: Developing and analysing a curriculum map in Occupational- and Environmental Medicine
Source: BMC Med Educ. 2010 Sep 14;10:60. doi: 10.1186/1472-6920-10-60 (PMC2944147; doi:10.1186/1472-6920-10-60)
Supplement: Additional file 1 — Translated questionnaire developed for this study. [file 1472-6920-10-60-S1.PDF]

Dear students,
please complete the following questionnaire!
Your feedback is very important and will help us to improve the quality of the course!

Thank you very much!

1) Age:____
2) Gender: ☐ m ☐ f
3) I am currently enroled in term___
4) Have you attended the lectures: ☐ yes ☐ no
5) If yes, how often?, If no, why not? ______________________________
6) The courses started on time: ☐ yes ☐ no
7) If no, please name the courses, that were not on time: ______________________
8) There was a lack of information regarding (please select one or more from the list):
   - ☐ Timetable
   - ☐ Exams
   - ☐ Learning objectives
   - ☐ Other, please name: ______________________

	5=totally agree, 1= totally disagree
9) I have learnt a lot during the course.	1○ 2○ 3○ 4○ 5○
10) The course content fitted to my previous knowledge	1○ 2○ 3○ 4○ 5○
11) The teachers motivated me to follow the course.	1○ 2○ 3○ 4○ 5○
12) The teachers motivated me to actively participate in the course.	1○ 2○ 3○ 4○ 5○
13) The course was practical.	1○ 2○ 3○ 4○ 5○
14) The learning objectives have been clearly defined.	1○ 2○ 3○ 4○ 5○
15) My personal learning objectives became clear.	1○ 2○ 3○ 4○ 5○
16) The teacher had a profound expertise.	1○ 2○ 3○ 4○ 5○
17) There were enough opportunities to ask questions.	1○ 2○ 3○ 4○ 5○
18) I realized what I know/don't know.	1○ 2○ 3○ 4○ 5○
19) The content was well structured.	1○ 2○ 3○ 4○ 5○
20) I was motivated to self-study.	1○ 2○ 3○ 4○ 5○
21) The teaching was according to the learning objectives.	1○ 2○ 3○ 4○ 5○
22) The learning objectives were adequate.	1○ 2○ 3○ 4○ 5○

23) I could have achieved the learning objectives in self-study.	1 O 2 O 3 O 4 O 5 O
24) I felt hindered by external conditions.	1 O 2 O 3 O 4 O 5 O
25) If applicable, please describe these external conditions.	1 O 2 O 3 O 4 O 5 O
26) I had fun during the course.	1 O 2 O 3 O 4 O 5 O
27) The final exam was adequate.	
	1=very good, 6=very bad
28) I grade the lectures as follows:	1 O 2 O 3 O 4 O 5 O 6 O
29) I grade the seminars as follows:	1 O 2 O 3 O 4 O 5 O 6 O
30) I grade the online cases as follows:	1 O 2 O 3 O 4 O 5 O 6 O

31) Comments
