# Supplementary material for: Developing and analysing a curriculum map in Occupational- and Environmental Medicine
Source: BMC Med Educ. 2010 Sep 14;10:60. doi: 10.1186/1472-6920-10-60 (PMC2944147; doi:10.1186/1472-6920-10-60)
Supplement: Additional file 2 — Table S1. [file 1472-6920-10-60-S2.DOC]

**Table 1 - Summary of the curriculum structure, evaluation and learning objectives of the courses**

| **Course (year)** | **Setting** | **Topics** | **Attendance** | **Rating* Mean ±SD** | **Learning  objectives** | **catalogue entries** |
| --- | --- | --- | --- | --- | --- | --- |
| Lecture OM  (3^rd^) | 6 voluntary lectures (8.15 - 9.00 am) | - Basic principles  - OM in medical service, infections, occupational   health and safety, psychic aspects  - Physical stress and strain  - Respiratory diseases, skin diseases  - Work-related cancer | 12 of 211  (range: 3-25) | 3.3 (1.187) (n=35) | 6.5 (per lecture)  (range: 4-9) | 8.5 (per lecture)  (range: 5-16) |
| Tutorial OM  (3^rd^) | 2 obligatory tutorials (1.5 hrs) in groups of 8 | Paper cases and each student giving a presentation, different topics covered in each tutorial depending on the tutor | 8 of 8 | 3.5 (1.379) | 8.0 (per tutorial)  (range: 5-11) | 11.5 (per tutorial)  (range: 5-16) |
| Online cases OM (3^rd^) | 2 of 6 obligatory | - Chromat exposure  - Lead intoxication  - Radiation protection  - Needle stick injury  - Tuberculosis, occupational disease  - Occupational asthma |  | 2.3 (0.993) | 3.1  (range: 2-5) | 6.5 (per case)  (range: 2-12) |
| OM exam | 30 MC questions |  |  |  | 1 (per question) | 1 (per question) |
| Lecture EM  (4^th^) | 6 voluntary lectures  (3.00 - 4.30 pm) | - Chemistry in EM, noise  - Electromagnetic fields  - Environmental tobacco smoke, particulate matter  - Ozone. aeriform pollutants  - Environmental syndromes (MCS)  - Allergic diseases in children | 11 of 223  (range: 4-29) | 2.9 (1.088) (n=16) | 4.3 (per lecture)  (range: 3-11) | 6.8 (per lecture)  (range: 3-11) |
| Online cases EM (4^th^) | 1 case obligatory | - Lead exposure |  | 2.2 (0.917) | 2 (per case) | 4 (per case) |
| EM exam | 30 MC questions |  |  |  | 1 (per question) | 1 (per question) |

* (grades from 1=very good to 6= very bad)
